# Identification of hub genes in the subacute spinal cord injury in rats

**DOI:** 10.1186/s12868-022-00737-5

**Published:** 2022-08-27

**Authors:** Lei Yan, Jiawei Fu, Xiong Dong, Baishen Chen, Hongxiang Hong, Zhiming Cui

**Affiliations:** The Second Affiliated Hospital of Nantong University, No.6, North Road, 226000 Haierxiang, Nantong, Jiangsu People’s Republic of China

**Keywords:** Spinal cord injury, WGCNA, PPI, Bioinformatics analysis

## Abstract

**Background:**

Spinal cord injury (SCI) is a common trauma in clinical practices. Subacute SCI is mainly characterized by neuronal apoptosis, axonal demyelination, Wallerian degeneration, axonal remodeling, and glial scar formation. It has been discovered in recent years that inflammatory responses are particularly important in subacute SCI. However, the mechanisms mediating inflammation are not completely clear.

**Methods:**

The gene expression profiles of GSE20907, GSE45006, and GSE45550 were downloaded from the GEO database. The models of the three gene expression profiles were all for SCI to the thoracic segment of the rat. The differentially expressed genes (DEGs) and weighted correlation network analysis (WGCNA) were performed using R software, and functional enrichment analysis and protein–protein interaction (PPI) network were performed using Metascape. Module analysis was performed using Cytoscape. Finally, the relative mRNA expression level of central genes was verified by RT-PCR.

**Results:**

A total of 206 candidate genes were identified, including 164 up-regulated genes and 42 down-regulated genes. The PPI network was evaluated, and the candidate genes enrichment results were mainly related to the production of tumor necrosis factors and innate immune regulatory response. Twelve core genes were identified, including 10 up-regulated genes and 2 down-regulated genes. Finally, seven hub genes with statistical significance in both the RT-PCR results and expression matrix were identified, namely Itgb1, Ptprc, Cd63, Lgals3, Vav1, Shc1, and Casp4. They are all related to the activation process of microglia.

**Conclusion:**

In this study, we identified the hub genes and signaling pathways involved in subacute SCI using bioinformatics methods, which may provide a molecular basis for the future treatment of SCI.

## Introduction

Spinal cord injury (SCI) is a serious complication of spinal fracture, in which the spinal cord or cauda equina is damaged to different degrees due to the displacement of the vertebral body or the intrusion of bone fragments into the spinal canal. It is a common trauma in clinics and has the characteristics of high incidence and disability rate. SCI is the main cause of long-term physical impairment and disability, which is a huge burden on the quality of life of patients and the medical system [1–4]. Despite significant advances in the state of the art of medical care in spinal surgery, there are currently no effective treatment options for this neurological disorder, mostly limited to supportive measures [5–7]. Traumatic SCI is divided into primary injury and secondary injury. Primary injury refers to mechanical direct injury to the spinal cord [8]; Secondary injury refers to the subsequent pathological reaction caused by direct phases. Secondary injury includes three stages: acute, subacute, and chronic injury. Acute secondary injury occurs within 0–48 h of injury and is initially characterized by increased calcium influx, ion imbalance, lipid peroxidation, free radical production, inflammation, and edema [9]. The stage of subacute secondary injury occurs two days to two weeks after injury and is characterized by neuronal apoptosis, axonal demyelination, Waller degeneration, axonal remodeling, and glial scar formation [9]. Over time, subacute lesions developed into chronic secondary lesions characterized by glial scar maturation, capfsular formation, and axonal dieback [10].

The most important stage in the pathophysiological process of SCI involves a secondary injury caused by neuroinflammation in the lesion area, accompanied by abnormal molecular signals, vascular changes, and secondary cell dysfunction, which is uncontrolled and destructive cascade [11–14]. Gene expression and signal pathways of that inflammatory cascade in subacute injury are complex [15]. During this period, the inflammatory cascade has dual effects, not only aggravating the tissue cell damage but also serving as an important promoting factor for the plastic change after SCI [16–18]. How to balance these dual effects is very important for the effect of intervention and treatment. Therefore, the relevant molecular mechanisms of inflammation in the subacute phase after SCI are worthy of in-depth study.

Bioinformatics is an emerging interdisciplinary subject that combines molecular biology and information technology, which opens up a new way for the diagnosis and treatment of human diseases [19]. Gene chip, as an emerging technology, has been used for efficient and large-scale access to biological information and can widely collect disease expression profile data. Some scholars have unveiled the activation pathways and molecular targets during acute or chronic SCI by identifying differentially expressed genes (DEGs) using microarray or RNA sequencing analysis [20–25]. However, the sequencing analysis for subacute SCI is relatively rare. In this paper, the bioinformatics tools are used to analyze the data from the sham operation group and SCI group of GSE20907, GSE45006, GSE45550 in the common gene chip databases, aiming to identify the key biomarkers of abnormally expressed genes in subacute SCI and provide targets for the diagnosis and treatment of subacute SCI.

## Materials and methods

### Download expression matrix data

**Fig. 1:**
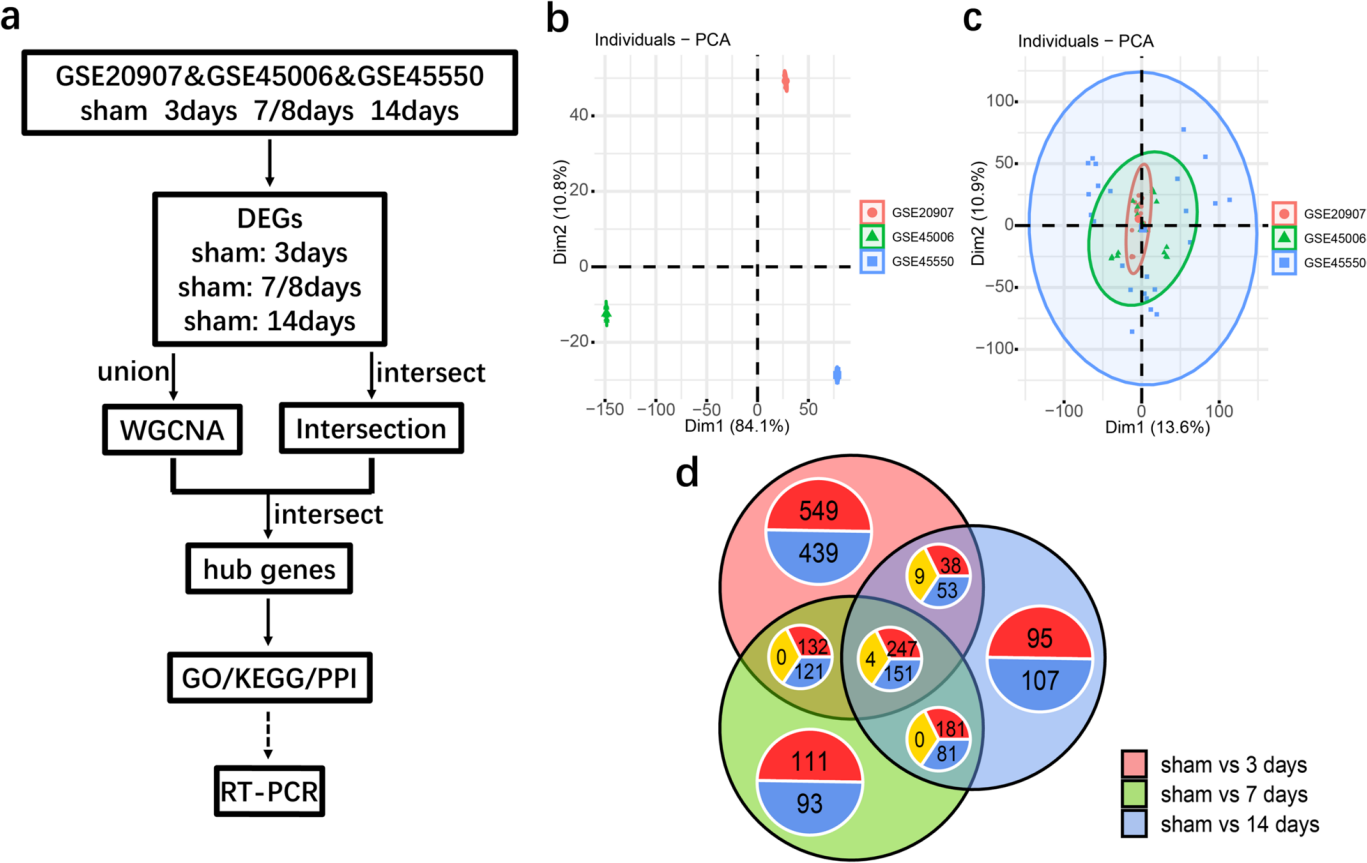
Research roadmap and data preprocessing. **a** Research roadmap. **b** PCA chart of three data sets without removing batch effect. **c** PCA chart of three data sets after removing batch effect. **d** Venn diagram of DEGs in sham operation group and 3 d, 7 d, and 14 d groups after subacute SCI. In each annotation circle, Red represented the number of up-regulated genes, Blue represented the number of down-regulated genes, and Yellow represented the number of genes with the opposite trend in the intersection set. Principal component analysis, PCA

Expression matrices of GSE20907, GSE45006, and GPL45550 were downloaded from the GEO database (https://www.ncbi.nlm.nih.gov/geo/). The ages of rats in the three data sets were about 9–11 weeks. GSE20907 [26] microarray data were based on the GPL6247 platform by performing a T9-T10 laser axonotomy in female Long Evans rats, followed by a moderate spinal contusion injury by dropping 10 g of the rod from a height of 25 mm using NYU Impactor. GSE45006 [27] microarray data were based on the GPL1355 platform and operated on female Wistar rats for T6-T8 laminectomy and moderate to severe impact compression damage using a 35 g Walsh clip for 1 min. GSE45550 [28] microarray data were based on the GPL1355 platform by performing dorsal laminectomy in the thoracic vertebrae T7-T9 of female Sprague Dawley rats, and the contusion was generated by dropping 10 g cylinders onto the T8 segment of the spinal cord from a height of 25 mm. Four groups of data from sham, on the 3rd, 7th, and 14th day after operation were extracted from each expression matrix for analysis. Information on these data sets is shown in Table 1. The experimental strategy in this paper is shown in Fig. 1-a.

**Table 1:** Number of samples per group contained in each dataset

Group GSE	Sham	3 days	7 days	14 days
GSE20907	8	4	4	2
GSE45006	4	4	4	4
GSE45550	6	6	6	6

### Data preprocessing and identification of DEGs

The expression matrix was subjected to batch effect elimination and batch normalization using R software (version 4.1.0; https://www.r-project.org/) and R-package SVA [29]. The Limma package in R (Limma;http://www.bioconductor.org/packages/release/bioc/html/limma.html) was used to identify DEGs by comparing expression values between sham and subacute SCI. The corresponding P-value of the gene symbols after the t-test was used. The adjusted P < 0.05 and |logFC|> 1 were used as the selection criteria.

### Weighted correlation network analysis (WGCNA)

WGCNA (v1.61; https://cran.r-project.org/web/packages/WGCNA/index.html) is a tool [30] for constructing gene co-expression networks and identifying gene clusters or modules. Thus, the WGCNA integration algorithm R (v3.4.1) was used to analyze highly relevant native gene clusters or modules for subacute SCI. There were ≥ 10 cutoff genes, cutting height = 0.85, Z‐ score ≥ 5, and stability-related stability correlation P ≤ 0.05 in this study. The connection of nodes (genes) between the two was used to calculate the data set, and genes with the correlation coefficient < 0.5 were excluded. The conservation status of WGCNA module and the traits related characteristics were analyzed.

### Time dynamic clustering analysis

To characterize the dynamics of subacute SCI gene expression, R-package Mfuzz[31] (v2.1; http://cran.r-project.org/web/packages/Mfuzz/index.html) was used for temporal dynamic cluster analysis. Fuzzy C-means clustering analysis, the core algorithm of Mfuzz, can aggregate genes with similar expression patterns. The differential genes were divided into four different clusters according to the expression pattern of subacute SCI. The cluster-score of the gene indicated the similarity of each cluster.

Functional enrichment analysis.

The KEGG/GO analysis was performed using the Metascape website (http://www.metascape.org/) [32].

### Protein–protein interaction network (PPI)

The PPI analysis was performed using the Metascape website (http://www.metascape.org/) [32]. The connectivity (degree) and hub nodes (genes) in PPI [33] were obtained using scale-free property to obtain. And MCODE algorithm was applied to this network, and GO enrichment analysis was applied to each MCODE network, each MCODE network being assigned a unique shape. For the hub nodes, the size of the shape represented the value of MCODE-degree. The results of PPI were imported into Cytoscape software (v3.9.0; http://www.cytoscape.org/) and further analyzed in combination with the results of temporal dynamic clustering analysis.

### Comparison of expression of candidate genes and real-time polymerase chain reaction (RT-PCR)

The heat map was made using R-packages ComplexHeatmap (v3.1; https://cran.r-project.org/web/packages/ComplexHeatmap/index.html) and GGplots (v3.0; https://cran.r-project.org/web/packages/ggplots/index.html) to compare the expression levels of candidate genes.

The hub genes were selected for RT-PCR. Female Sprague Dawley rats (6–8 weeks of age, average weight 210 g) were acquired from the Laboratory Animal Center of Nantong University (Nantong, China). Rats were provided with normal food and water and housed at 20–26 ℃ under 55%-65% humidity with a 12 h/12 h artificial diurnal cycle. Rats underwent general anesthesia (20 ml/kg) by intraperitoneal injection of avertin (2, 2, 2-tribromoethanol, Sigma-Aldrich) in 0.9% saline solution and were injured by impact compression using a 35 g Walsh clip for 1 min at thoracic level T7-T9 and sacrificed (CO2 asphyxia) for spinal cord tissue removal 3, 7, and 14 days after SCI. Five rats in each group. Total RNA was isolated using TRIZOL reagent (Invitrogen, Thermo Fisher Scientific, Inc.) and reverse transcribed into cDNA according to the manufacturer's instructions. RT-PCR was performed using SYBR green dye(Takara) in a thermal cycler under the following parameters: initial denaturation step at 95 ℃ for 30 min; 40 cycles at 95 ℃ for 5 s; And 60 ℃ for 30 s. The complete experimental procedure was performed on each sample in duplicate. The mRNA primers are shown in Table 2.

**Table 2:** Hub genes primers used in this study

Gene	Name	Forward primer	Reverse primer
Itgb1	Integrin subunit β1	GGAGATGGGAAACTTGGTGGT	TAGAGTTTCCAGACAGTGTGCC
Fcgr2b	Fc gamma receptor IIb	TCCAAGCCTGTCACCATCAC	TGGCAGCTACAGCAATTCCA
Ptprc	Protein tyrosine phosphatase receptor type C	TGACTCGGAAGAAACCAGCA	AGTCTGCTTTCCTTCTCCCC
S100a4	S100 calcium binding protein A4	CAAATACTCAGGCAACGAGGG	CACATCATGGCAATGCAGGAC
Cd63	Cd63	GGGGCCTGCAAAGAGAACTA	TTGTCCAAAATGGTGGCCGT
Lgals3	Galactose-specific lectin 3	AGGCTCCTCCTAGTGCCTAT	CCTCCAGGCAAGGGCATATC
Lamc1	Laminin subunit gamma 1	TCTTGGACCTTACAGCCCGT	GTGCACACCACTTCCTTTGTC
Vav1	Guanine nucleotide exchange factor 1	AGGAGTGTCTGGGAAGGGTG	AGTTCCACAATGTCCCCAGG
Shc1	Shc adaptor protein 1	TGTGAATCAGAGAGCCTGCC	TCATCCCAAGCTGAGCCATC
Casp4	Cysteine peptidase 4	GTGACAAGCGCTGGGTTTTT	TCTGCACAGCCTTGTGAACT
Mapk12	Mitogen-activated protein kinase 12	CCATTCATGGGCACTGACCT	GTCATCTCACTGTCCGCCTG
Vegfa	Vascular endothelial growth factor a	AAGGCGCGCAAGAGAGC	AATTGGACGGCAATAGCTGC

### Data analysis

SPSS 22.0 software (IBM, Armonk, NY, USA) was used for all statistical analyses. If the data both showed normality and homogeneity of variance, they were expressed as mean and standard deviation. The Unpaired Student’s t-test was used for inter-group comparison. If the data were not showed normality or homogeneity of variance, the quantitative variables were expressed as the median of the ranges and compared between groups using the Mann–Whitney Wilcoxon test, respectively. All significance levels were set to P < 0.05 and plotted using GraphPad Prism 6 (GraphPad Software Inc., CA, USA).

## Results

### Data preprocessing and identification of DEGs

To eliminate the batch effect of merging different datasets, microarray results from GSE20907, GSE45006, and GSE45550 were batch corrected and normalized using PCA (Fig. 1b, c), and DEGs were selected by comparing the surgical group and the subacute SCI groups (3 days /7 days /14 days) using R-package Limma. The Venn diagram was drawn using R software (Fig. 1d). There were 2414 genes in the union of DEGs detected, and 402 genes in the intersection of DEGs detected. In the sham operation group, there were 771 down-regulated genes and 972 up-regulated genes compared with the 3-day group, 477 down-regulated genes and 677 up-regulated genes compared with the 7-day group, and 398 down-regulated genes and 571 up-regulated genes compared with the 14-day group.

### WGCNA of the union of DEGs

**Fig. 2:**
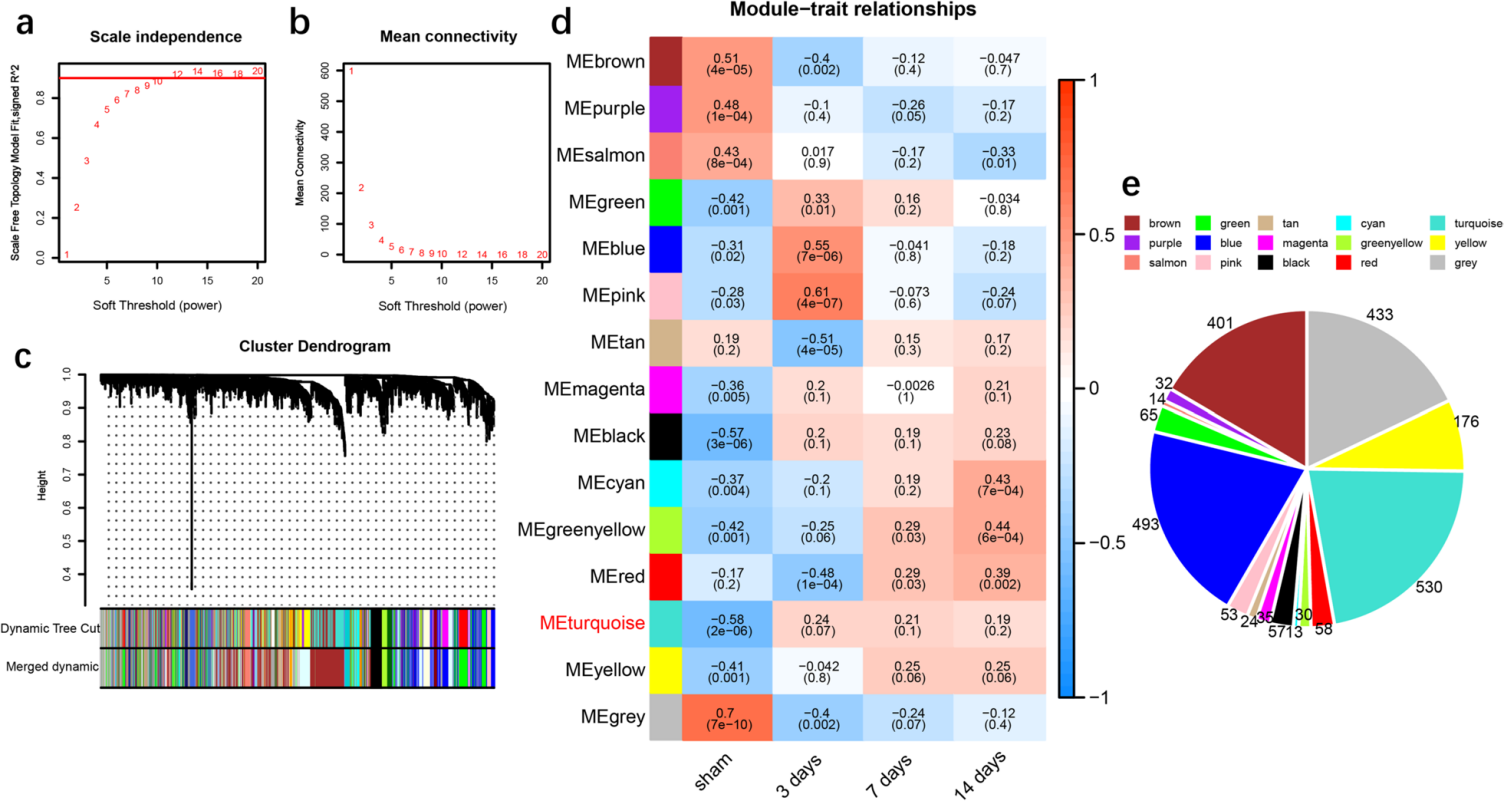
WGCNA of the union of DEGs. **a** scale independence, **b** mean connectivity. The network topology analysis for adjacency matrix with different soft threshold power. Red numbers in the boxes indicate the soft thresholding power corresponding to the correlation coefficient square value(y-axis). **c** consensus module dendrogram was produced by clustering of 2414 genes with a variation coefficient of expression > 0.1, based on the criteria of correlation coefficient square of eigengenes above 0.85, soft threshold power of 9, the number of genes > 10, and cut height = 0.95. **d** Module-trait associations. Each row corresponds to a module trait gene, and each column corresponds to a trait. Red indicated a positive correlation between modular trait genes and traits, and blue indicated a negative correlation. Each cell contains the correlation coefficient Rho and the P-value in parentheses. **e** Pie chart of the number of genes in modules, each color representing each module. WGCNA, weighted correlation network analysis

The union of 2414 DEGs was taken as the expression matrix and used as the input data for network construction. According to the rules of scale-free networks, the larger the correlation coefficient is, the more significant the scale-free property of the network is. According to the prerequisite of the approximate scale-free topology processed by WGCNA, the soft threshold power of the adjacency matrix is 9, and the standard that the square of the intrinsic gene correlation coefficient is greater than 0.85 is taken as the standard for module identification (Fig. 2a). At this time, the average connectivity was 1, indicating that the gene module was constructed according to the approximate scale-free topology standard (Fig. 2b). After the soft threshold was determined, the expression matrix of the differential genes was converted into an adjacency matrix, a topology matrix, and a dissimilarity matrix between genes. On this basis, the hierarchical clustering method is used for gene clustering, and the dynamic cutting algorithm is used for module identification of the system clustering tree. Fifteen different co-expression modules were obtained and expressed in different colors (Fig. 2c). These modules were correlated to clinical features and modules of continuous correlation were found (Fig. 2d). From the results, we could see that the correlation coefficients of the three subgroups of subacute SCI in the Turquoise module were gradually decreased (Rho_3_ = 0.24, Rho_7_ = 0.21, Rho_14_ = 0.19), suggesting that the genes in this module had a continuous correlation with subacute SCI. Figure 2e shows the number of genes in each module, in which the Turquoise module contained 530 genes.

### Functional enrichment analysis of candidate genes

**Fig. 3:**
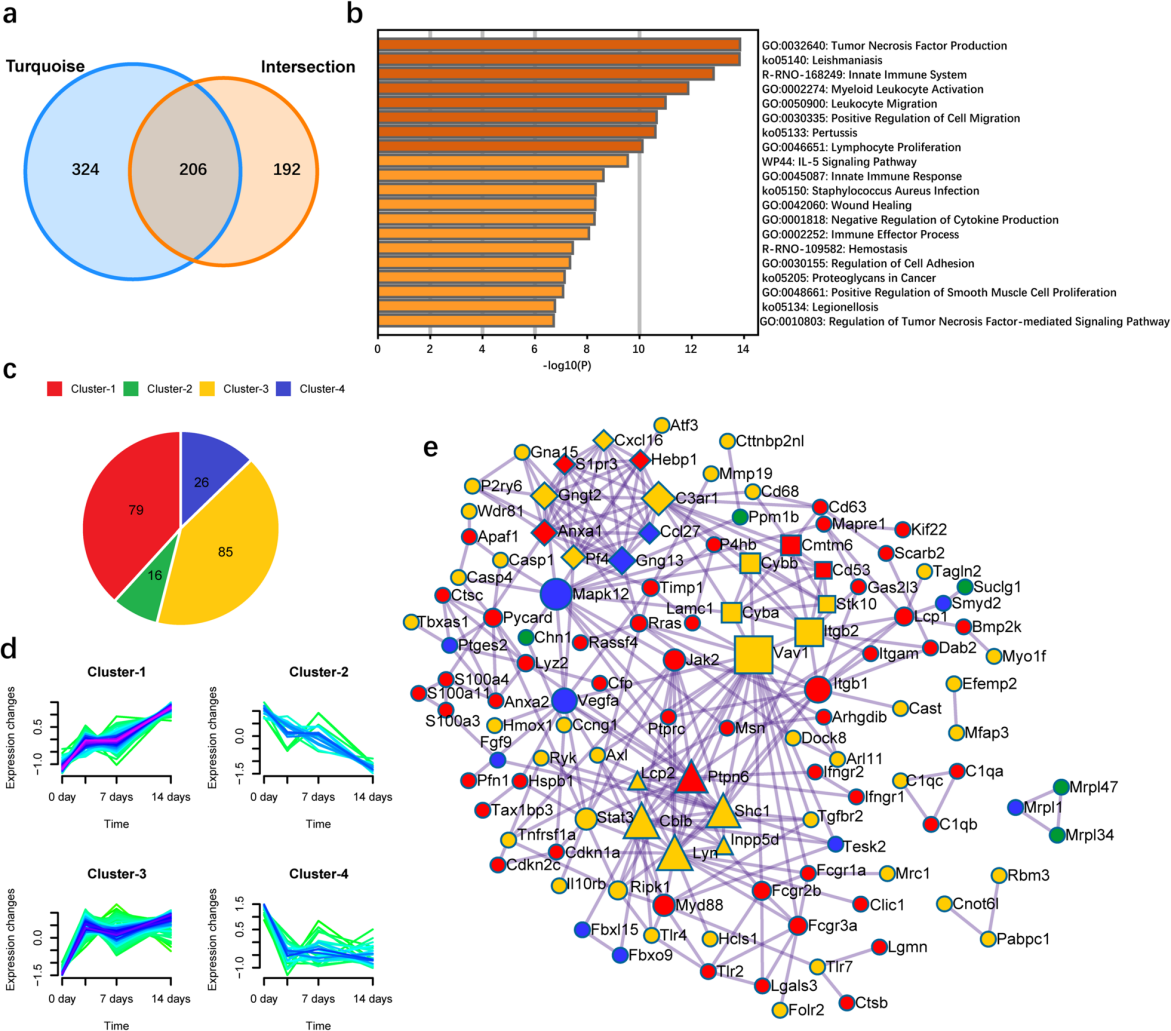
Functional enrichment analysis and PPI analysis of candidate genes. **a** Venn diagram analysis of genes between the Turquoise module and the Intersection. **b** Functional enrichment analysis for 206 candidate genes. **c** Pie chart of the number of genes of four clusters in time-dynamic clustering analysis of 206 candidate genes. Four different colors represent different clusters. **d** Change trend of the relative expression level of four clusters in time-dynamic clustering analysis of 206 candidate genes. **e** PPI analysis of candidate genes. Four different colors represent different clusters in the temporal dynamic clustering analysis MCODE algorithm was applied to this network, and GO enrichment analysis was applied to each MCODE network, each MCODE network being assigned a unique shape. For the hub nodes, the size of the shape represented the value of MCODE-degree. PPI, protein–protein interaction

398 genes in the Intersection (402 genes exclude 4 genes with different trends) and 530 genes in the Turquoise module were crossed again to obtain 206 candidate genes (Fig. 3a). The GO function and KEGG pathway of candidate genes were annotated with Metascape, and the top 20 results were shown in Fig. 3b and Table 3. We found that these genes were mainly associated with 12 GO Biological Processes, including Tumor Necrosis Factor Production, Myeloid Leukocyte Activation, Leukocyte Migration, Positive Regulation of Cell Migration, Lymphocyte Proliferation, Innate Immune Response, Wound Healing, Negative Regulation of Cytokine Production, Immune Effector Process, Regulation of Cell Adhesion, Positive Regulation of Smooth Muscle Cell Proliferation, and Regulation of Tumor Necrosis Factor-mediated Signaling Pathway; It was associated with five KEGG Pathways, including Leishmaniasis, Pertussis, Staphylococcus Aureus Infection, Proteoglycans in Cancer, and Legionellosis; It was associated with two Reactome Gene Sets, including Innate Immune System and Hemostasis; Related to one WikiPathways for IL-5 signaling pathway.

**Table 3:** The top 20 results of the GO function and KEGG pathway of candidate genes

Category	GO	Description	Genes	Log(q-value)
GO Biological Processes	GO:0032640	tumor necrosis factor production	Tspo Hspb1 Jak2 Ptprc Stat3 Tnfrsf1a Tlr4 Cybb Cyba Gpnmb Ifngr1 Ptpn6 Pycard Myd88 Ripk1 Axl Tlr2 Pf4 Clec4a3 Ly96	− 10
KEGG Pathway	ko05140	Leishmaniasis	Itgb1 Jak2 Itgam Tlr4 Mapk12 Cyba Ifngr1 Ptpn6 Fcgr1a Myd88 Fcgr3a Itgb2 Tlr2 Ifngr2	− 10
Reactome Gene Sets	R-RNO-168249	Innate Immune System	Cd53 Ptprc Ctsc Cd63 Tlr4 C1qb Anxa2 Mapk12 Lgmn Pygl Apaf1 Cyba Lyn Lgals3 C3ar1 Shc1 Gmfg Ptpn6 Lcp2 Cd68 Fcgr2b Serpinb1a Folr2 C1qa Cfp Myd88 Fcgr3a Nkiras1 Itgb2 Tlr2 Ptges2 Cmtm6 Tlr7 C1qc S100a11	− 9.4
GO Biological Processes	GO:0002274	myeloid leukocyte activation	Hmox1 Jak2 Itgam Casp1 Ctsc Tlr4 Lyn Tgfbr2 Ifngr1 Lcp2 Pycard Fcgr2b C1qa Myd88 Fcgr3a Tnip2 Itgb2 Tlr2 Myo1f Ifngr2 Btk	− 8.5
GO Biological Processes	GO:0050900	leukocyte migration	B4galt1 Itgb1 Itgam Vav1 Ninj1 Anxa1 Stk10 P2rx4 Lgmn Lyn Msn Lgals3 Vegfa C3ar1 Pycard Folr2 Myd88 Itgb2 Tlr2 Pf4 Ccl27 St3gal4 Cxcl16 Dock8	− 7.9
GO Biological Processes	GO:0030335	positive regulation of cell migration	Hmox1 Hspb1 Itgb1 Jak2 Ptprc Stat3 Anxa1 Fgf9 Tlr4 P2rx4 Lgmn Pfn1 Dab2 Lyn Tgfbr2 Lgals3 Vegfa C3ar1 Gpnmb P2ry6 Pycard Flna Tlr2 Myo1f Rras Ccl27 S100a11 Cxcl16 Dock8	− 7.6
KEGG Pathway	ko05133	Pertussis	Itgb1 Itgam Casp1 Tlr4 C1qb Mapk12 Pycard C1qa Myd88 Itgb2 C1qc Ly96	− 7.6
GO Biological Processes	GO:0046651	lymphocyte proliferation	Ptprc Itgam Anxa1 Tlr4 Inpp5d Lyn Msn Tgfbr2 Lgals3 Laptm5 Gpnmb Cdkn1a Ptpn6 Cblb Pycard Fcgr2b Myd88 Pura Itgb2 Btk Dock8	− 7.1
WikiPathways	WP44	IL-5 signaling pathway	Jak2 Itgam Stat3 Vav1 Alox5ap Lyn Shc1 Ptpn6 Hcls1 Itgb2 Btk	− 6.7
GO Biological Processes	GO:0045087	innate immune response	Jak2 Vav1 Casp1 Anxa1 Tlr4 C1qb Cybb Cyba Lyn Lgals3 Ptpn6 Slc15a3 Pycard Serpinb1a Mrc1 Capg C1qa Cfp Fbxo9 Myd88 Cdc42ep2 Tlr2 Tnfaip8l2 Myo1f Tlr7 Ifitm3 C1qc Cxcl16	− 5.9
KEGG Pathway	ko05150	Staphylococcus aureus infection	Itgam C1qb C3ar1 Fcgr2b Fcgr1a C1qa Fcgr3a Itgb2 C1qc	− 5.6
GO Biological Processes	GO:0042060	wound healing	B4galt1 Hmox1 Hspb1 Itgb1 Jak2 Anxa1 Tlr4 Anxa2 Lyn Tgfbr2 Vegfa Cdkn1a Timp1 Ptpn6 Flna Il10rb Fcgr3a Lcp1 Axl Pf4 St3gal4 Clic1	− 5.6
GO Biological Processes	GO:0001818	negative regulation of cytokine production	Tspo Hmox1 Ppm1b Ptprc Anxa1 Tnfrsf1a Tlr4 Inpp5d Laptm5 Gpnmb Ptpn6 Pycard Fcgr2b Serpinb1a Axl Tlr2 Clec4a3 Btk	− 5.6
GO Biological Processes	GO:0002252	immune effector process	Hmox1 Ptprc Itgam Stat3 Vav1 Anxa1 Ctsc Tlr4 C1qb Inpp5d Lyn Lgals3 Laptm5 Ptpn6 Pycard Fcgr2b Fcgr1a C1qa Cfp Myd88 Lcp1 Itgb2 Tlr2 Myo1f C1qc Btk	− 5.4
Reactome Gene Sets	R-RNO-109582	Hemostasis	Itgb1 Jak2 Cd63 P2rx4 Inpp5d Anxa2 Lyn Slc7a7 Vegfa Shc1 Gna15 Timp1 Ptpn6 Lcp2 Kif22 Flna Itgb2 Pf4 Dock8 Gng13 Gngt2	− 4.9
GO Biological Processes	GO:0030155	regulation of cell adhesion	Hspb1 Itgb1 Jak2 Ptprc Vav1 Ninj1 Anxa1 P4hb Cd63 Dab2 Lyn Tgfbr2 Lgals3 Vegfa Laptm5 Gpnmb Ptpn6 Cblb Pycard Efemp2 Flna Itgb2 Tnfaip8l2 Myo1f Rras St3gal4 Dock8 Coro1c	− 4.9
KEGG Pathway	ko05205	Proteoglycans in cancer	Itgb1 Stat3 Cd63 Tlr4 Mapk12 Msn Vegfa Cdkn1a Ptpn6 Cblb Hcls1 Flna Tlr2 Rras	− 4.7
GO Biological Processes	GO:0048661	positive regulation of smooth muscle cell proliferation	Hmox1 Jak2 Fgf9 Tlr4 Cyba Tgfbr2 Vegfa C3ar1 Shc1 P2ry6 Myd88	− 4.6
KEGG Pathway	ko05134	Legionellosis	Itgam Casp1 Tlr4 Apaf1 Pycard Myd88 Itgb2 Tlr2	−4.4
GO Biological Processes	GO:0010803	regulation of tumor necrosis factor-mediated signaling pathway	Casp1 Tnfrsf1a Laptm5 Casp4 Pycard Nkiras1 Ripk1	− 4.4

### Temporal dynamic clustering analysis and PPI analysis of candidate genes

Temporal dynamic clustering analysis was performed on the candidate genes using R-package Mfuzz. As shown in Fig. 3c, d, the candidate genes were divided into four clusters with different expression modes. Cluster-1 represented 79 genes with continuous high expression over time after SCI, Cluster-2 represented 16 genes with continuous low expression over time after SCI, Cluster-3 represented 85 genes with high expression after SCI, and Cluster-4 represented 26 genes with low expression after SCI. PPI analysis of candidate genes was performed using the Metascape, and the results showed that 117 genes among the candidate genes served as hub nodes, as shown in Fig. 3e. There were 52 hub nodes in Cluster-1, 5 hub nodes in Cluster-2, 49 hub nodes in Cluster-3, and 11 hub nodes in Cluster-4. The top three network shapes for Log(q-value) values are Diamond, Rectangle, and Triangle. As shown in Table 4, the hub nodes are mainly related to the innate immune regulation of GO function. MCODE-1 is mainly related to the regulation of the GPCR signaling pathway, MCODE-2 is mainly related to the cross-endothelial migration of leukocytes, and MCODE-3 is mainly related to the Kit receptor signaling pathway and cytokine.

**Table 4:** GO enrichment analysis of MCODE network in PPI.MCODE algorithm was applied to this network, and GO enrichment analysis was applied to each MCODE network

Network	Shape	Gene	Go	Description	Log(q-value)
ALL	Ellipse	–	R-RNO-168249	Innate immune system	− 17.2
			GO:0050778	positive regulation of immune response	− 15.4
			GO:0002252	immune effector process	− 12.9
MCODE_1	Diamond	C3ar1 Ccl27 Pf4 S1pr3 Cxcl16 Hebp1 Gngt2 Anxa1 Gng13	R-RNO-418594	G alpha (i) signalling events	− 16.9
			R-RNO-500792	GPCR ligand binding	− 15.5
			R-RNO-388396	GPCR downstream signalling	− 14.7
MCODE_2	Rectangle	Itgb2 Cybb Cyba Vav1 Cd53 Cmtm6 Stk10	rno04670	Leukocyte transendothelial migration	− 7.2
			ko04670	Leukocyte transendothelial migration	− 7.2
			GO:0042554	superoxide anion generation	− 6.2
MCODE_3	Triangle	Shc1 Ptpn6 Inpp5d Lyn Cblb Lcp2	WP147	Kit receptor signaling pathway	− 11.4
			R-RNO-512988	Interleukin-3, Interleukin-5 and GM-CSF signaling	− 9.8
			R-RNO-21099	PECAM1 interactions	− 8.6

*PPI* protein–protein interaction

### Comparison of hub genes expression levels and RT-PCR verification

**Fig. 4:**
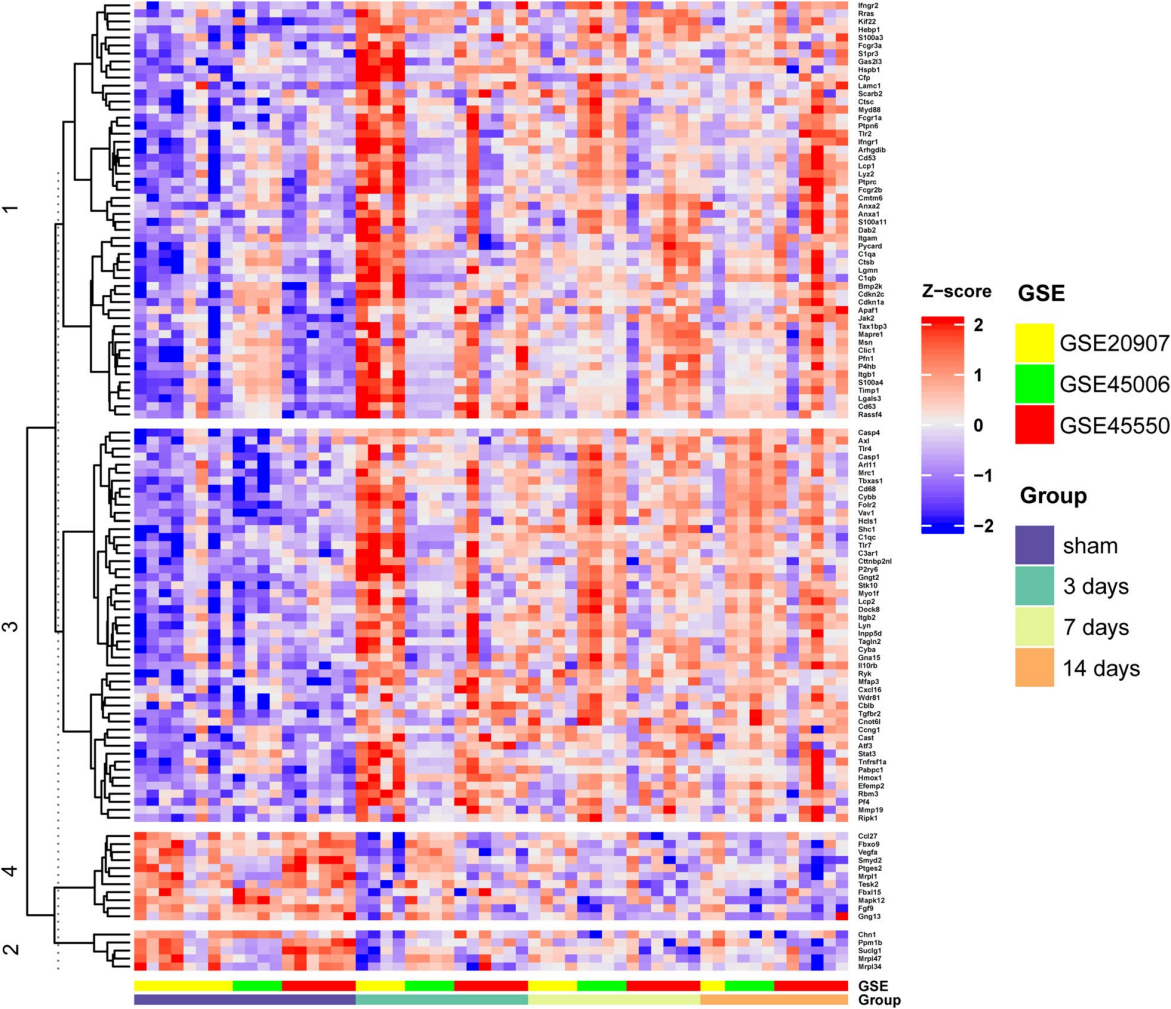
Heat map of 117 hub nodes in PPI. The numbers on the left represent clusters of time dynamic analysis. PPI, protein–protein interaction. PPI, protein–protein interaction

**Fig. 5:**
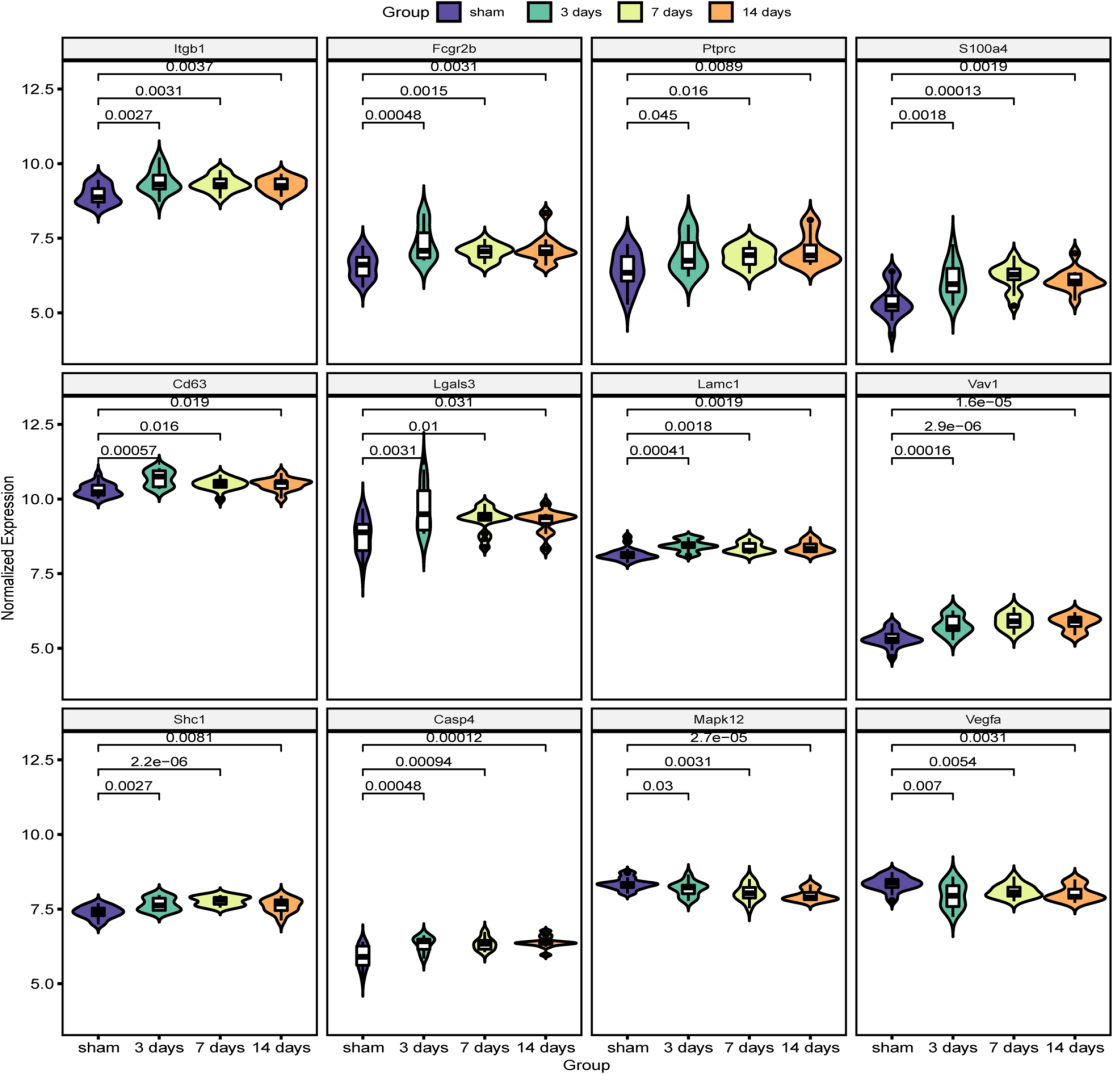
Violin plot of the normalized expression levels of 12 hub genes based on the combined expression matrix

**Fig. 6:**
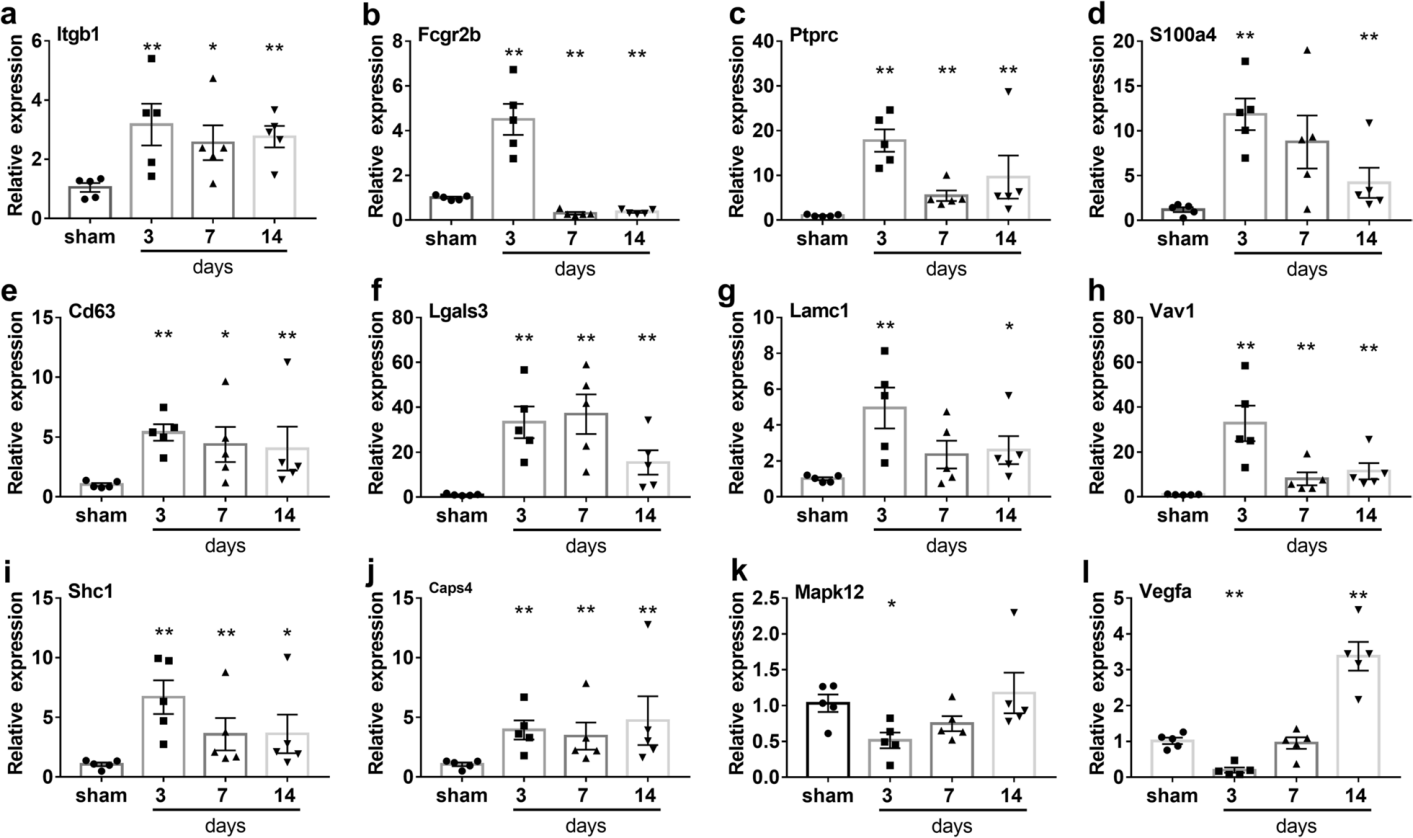
RT-PCR validation. **a**–**l** Relative expression levels of 12 hub genes. 5 samples per group in duplicate were analyzed using RT-PCR and summarized as mean average ± SE with P < 0.05. A pairwise comparison was made between the sham operation group and 3, 7, and 14 days after subacute SCI, a Mann–Whitney Wilcoxon's test was performed. *P < 0.05, **P < 0.01. RT-PCR, real-time polymerase chain reaction; SCI, spinal cord injury; SE, standard error

The 117 genes were produced into heat map as shown in Fig. 4, and the 12 hub genes selected according to the MCODE-degrees and the cluster-scores are shown in Table 5, which are Itgb1, Fcgr2b, Ptprc, S100a4, Cd63, Lgals3, Lamc1, Vav1, Shc1, Casp4, Mapk12, and Vegfa. Figure 5 shows the different expression levels of the 12 hub genes in the control and experimental groups in the combined data set. Figure 6 shows the results of hub genes validation in rats by RT-PCR. The relative expression levels of the seven central genes, Itgb1, Ptprc, Cd63, Lgals3, Vav1, Shc1, and Casp4, were consistent with the expression matrix. The relative expression level of Fcgr2b increased on the 3rd day after SCI but decreased on the 7th and 14th days. The relative expression levels of S100a4 and Lamc1 were statistically significant on the 3rd and 14th days after SCI, but not on the 7th day. The relative expression levels of Mapk12 and Vegfa decreased on the 3rd day after SCI, which was consistent with the expression matrix, but increased on the 7th and 14th days after SCI, which was inconsistent with the expression matrix.

**Table 5:** The MCODE-degrees and the cluster-scores of 12 hub genes

Gene	MCODE_degree	Cluster	Cluster_score	Up/down
Itgb1	12	1	0.708	up
Fcgr2b	7	1	0.691	up
Ptprc	5	1	0.746	up
S100a4	4	1	0.758	up
Cd63	4	1	0.420	up
Lgals3	3	1	0.791	up
Lamc1	3	1	0.762	up
Vav1	19	3	0.503	up
Shc1	16	3	0.589	up
Casp4	3	3	0.420	up
Mapk12	15	4	0.393	down
Vegfa	11	4	0.624	down

## Discussion

SCI leads to motor and sensory dysfunction, and the cascade of primary injury leads to the complex cascade of secondary injury events. Studies have shown that triggering immunoreaction in the subacute phase after spinal cord injury combined with rehabilitation training is more conducive to the recovery of spinal cord function [34]. As an animal with high homology to humans, low price, and easy feeding, rats have been widely used to make spinal cord injury models. In this study, gene expression profiles of GSE20907, GSE45006, and GSE45550 were combined for the first time to conduct DEGs analysis of the subacute SCI in rats. Although the differences among the SCI models with three gene expression profiles may lead to differences in signaling pathways and functional molecules, we assume that common and collective molecular mechanisms of injury may occur even in different SCI models, and identifying these mechanisms can provide new ideas for the treatment and prognosis judgment of SCI.

In this study, we first performed WGCNA using the union of DEGs. WGCNA has been well applied in biomedical research, and its analysis has mainly focused on specific phenotypes and co-expression modules. Genes in the same module are considered to be functionally related, with higher reliability and biological significance [35, 36]. Therefore, this analysis allows the identification of biologically relevant modules and core genes that can ultimately be used as biomarkers for SCI detection or treatment. The analysis results of WGCNA showed that the Turquoise module was considered to be the module most related to the subacute SCI (3 days to 14 days), and then intersected with the intersection of DEGs to obtain 206 candidate genes. At this time, the functional enrichment analysis and PPI analysis of these candidate genes were conducted to analyze the possible potential interactions and potentially significant molecular regulatory network mechanisms between DEGs-encoded proteins, and the results showed that the candidate genes were mainly related to the production of cellular inflammatory tumor necrosis factor and the innate immune regulatory response. Studies have shown that identification of various immune responses, including activation of the complement system, induction of innate and adaptive immune responses, and antibody production [37, 38] based on GO functional analysis is the most significantly upregulated biological process during acute SCI. This study also showed the same results during subacute SCI, indicating that inflammatory injury is still dominant in subacute SCI. The results of temporal dynamic clustering analysis showed that the expression patterns of 206 candidate genes were mainly Cluster 1 which continuously showed high expression over time and Cluster 3 which always maintained a high expression level and the expression level did not significantly change over time. Besides, the main hub nodes in PPI were Cluster 1 and Cluster 3 with up-regulated DEGs, while only five genes in Cluster 2 were the hub nodes. The Log(q-value) value of the hub nodes in MCODE-1 was the largest, indicating that the GPCR signaling pathway played a very important role in subacute SCI. The peptide energy system was the most abundant network for human ligand receptor-mediated signaling, which has been widely studied in inflammation. It has been shown that the GPCR ligand promotes neuroinflammation in central nervous system diseases [39], which supports our findings. It is well known that the cross-endothelial migration of leukocytes is an indispensable link in tissue inflammation, so the results in MCODE-2 also indicate this point. In addition, the Kit receptor and cytokine-related signaling pathways in MCODE-3 have been widely studied in inflammatory mechanisms, and it has been shown to play a role in the peripheral nervous inflammation mechanism [40].

According to MCODE-degree and cluster-score, twelve hub genes were screened and verified by RT-PCR. The results showed that the relative expression levels of seven hub genes, namely Itgb1, Ptprc, Cd63, Lgals3, Vav1, Shc1, and Casp4, were consistent with microarray hybridization. It is worth mentioning that all of them were related to the activation process of microglia. As is known to all, activated microglia are the key factors for the occurrence and development of SCI [41–43]. Itgb1 belongs to the family of adhesion molecules, and its signal transmission is bidirectional, including cell polarity change, regulation of movement, gene expression, and other mechanisms. The "internal–external" and "external-internal" signal regulation are closely related and affect each other [44]. Studies have shown that Itgb1 is widely expressed in the nervous system and may play a central role in physiological processes [45]. Moreover, abnormal expression of Itgb1 is related to neuropathic pain, inflammation, and malignant diseases due to peripheral nerve injury [46]. Meanwhile, a recent study has pointed out that the administration of anti-Itgb1 antibody (β1-Ab) in the subacute SCI successfully prevented glial scar formation and enhanced axonal regeneration [47]. Ptprc can be used as a specific marker for the activation of microglia [48]. After T-cell activation, Ptprc recruits and dephosphorylates SKAP1 and FYN, and dephosphorylates LYN, thereby regulating LYN activity. Cd63 acts as a cell surface protein in the activation regulation of the signaling cascade of cell development, activation, growth, and movement. Cd63 plays a role in the activation of Itgb1 and integrin signaling, leading to the activation of AKT, FAK/PTK2, and MAP kinases [49]. A recent study has shown that the expression of Cd63 in both plasma and spinal cord tissues is increased after SCI in mice [50]. Lgals3 is involved in central nervous system inflammation, including neutrophil activation and adhesion, chemical traction of mononuclear macrophages, regulation of apoptotic neutrophils, and activation of mast cells [51]. Some scholars have pointed out that Lgals3 plays an important role in regulating microglial activation and neuroinflammation, serving as a biomarker of neurodegenerative diseases [52]. Vav1 can mediate the Rho activation of JAK as a guanine nucleotide exchange factor of Rho family GTP enzymes and plays an important role in the development and activation of T cells and B cells [53]. Vav1 is highly expressed in the early reaction and regeneration stage of sciatic nerve injury and activates the Rac1 GTP enzyme to promote axonal regeneration of DRG neurons [54]. Shc1, a downstream target of tumor suppressor p53, is essential for the ability of stress-activated p53 to induce increased intracellular oxidants, cytochrome c release, and apoptosis. Although studies have suggested that Shc1 plays an important role in regulating microglia polarization [55], research on Shc1 in the nervous system is relatively rare. Casp4 is involved in the activation of inflammatory bodies and has been widely studied. An Alzheimer's disease study pointed out that Casp4 regulates microglia [56] in a way that increases the pro-inflammatory process. Our study showed that these seven hub genes could be used as potential targets for exploring the molecular mechanisms related to the development of subacute SCI over time and for balancing the injury and repair induced by SCI.

In addition, we also verified the mRNA relative expression levels of five other hub genes (Fcgr2b, S100a4, Lamc1, Mapk12, and Vegfa) in spinal cord tissues in the subacute SCI, although their results do not conform to the expression matrix. Fcgr2b is a low-affinity receptor in the Fc region of immunoglobulin γ complex, and its cytoplasmic domain contains an inhibitory motif (ITIM), which is the only inhibitory Fcg receptor. The results of a mouse experimental model with cerebral ischemia showed that the expression level of Fcgr2b of microglia/macrophage activated by inflammatory reaction remained unchanged [57]. but the research on Fcgr2b in the nervous system was relatively rare, the reason why the relative mRNA expression level of Fcgr2b increased in the early stage of subacute SCI but decreased in the advanced stage needs to be further explored. S100a4 is a calcium-binding protein. It plays a role in a variety of cellular processes, including motility, angiogenesis, cell differentiation, apoptosis, and autophagy [58]. Lamc1 belongs to the extracellular matrix glycoprotein family and is the major non-collagen component of the basement membrane. They are related to various biological processes, including cell adhesion, differentiation, migration, signal transduction, neurite outgrowth, and metastasis [59]. In this study, there was no statistical difference between the 7-day after subacute SCI group and the sham operation group possibly due to sample size. Mapk12 plays a vital role in cellular processes, involving inflammation, cell growth, cell differentiation and cell cycle action [60]. Vegfa is able to inhibit apoptosis and induce endothelial cell proliferation, promote cell migration, and axonal regeneration [61]. RT-PCR verification showed that their expression levels decreased when stimulated by inflammatory injury, but increased when the disease progressed due to regeneration and repair of the injury site. Further experimental studies are needed to explain the mechanism of why the expression matrix is inconsistent with RT-PCR verification. This is also a limitation of our study. In our study, the predictive results were mainly based on bioinformatics analysis, and the research sample was from rats but not humans, so clinical and relevant biological experiments are needed to further explore and prove the action mechanism of central genes. To sum up, our study provides some meaningful insights into the disease progression mechanisms and targeted therapeutic strategies for subacute SCI patients, and helps to develop new therapeutic strategies for SCI.

In this study, comprehensive bioinformatics analysis was performed on the gene expression profiles of rats at three different time points after subacute SCI. A total of 12 hub genes were identified and seven hub genes with statistical significance in both the RT-PCR results and the expression matrix were identified. The biological functions and pathways of the identified genes provide more detailed molecular mechanisms for understanding the disease progression of subacute SCI. In conclusion, we have identified the hub genes and signaling pathways involved in subacute SCI by using bioinformatics methods, which may provide a molecular basis for the future treatment of SCI.

## Data Availability

All data generated or analyzed during this study are included in this published article and its supplementary information files.
